# Reduced expression of CD109 in tumor-associated endothelial cells promotes tumor progression by paracrine interleukin-8 in hepatocellular carcinoma

**DOI:** 10.18632/oncotarget.8787

**Published:** 2016-04-18

**Authors:** Bo-Gen Ye, Hui-Chuan Sun, Xiao-Dong Zhu, Zong-Tao Chai, Yuan-Yuan Zhang, Jian-Yang Ao, Hao Cai, De-Ning Ma, Cheng-Hao Wang, Cheng-Dong Qin, Dong-Mei Gao, Zhao-You Tang

**Affiliations:** ^1^ Liver Cancer Institute and Zhongshan Hospital, Fudan University, Shanghai 200032, China; ^2^ Key Laboratory of Carcinogenesis and Cancer Invasion, Ministry of Education, Shanghai 200032, China; ^3^ General Surgery, Changhai Hospital, Second Military Medical University, Shanghai 200433, China; ^4^ The First Affiliated Hospital of Wenzhou Medical University, Wenzhou 325000, China

**Keywords:** CD109, hepatocellular carcinoma, interleukin-8, TGF-β, tumor-associated endothelial cells

## Abstract

Tumor-associated endothelial cells (TEC) directly facilitate tumor progression, but little is known about the mechanisms. We investigated the function of CD109 in TEC and its clinical significance in hepatocellular carcinoma (HCC). The correlation between CD109 expressed on tumor vessels and the prognosis after surgical resection of HCC was studied. The effect of human umbilical vein endothelial cells (HUVEC) with different CD109 expression on hepatoma cell proliferation, migration, and invasion was compared in co-culture assay. Associated key factors were screened by human cytokine antibody array and validated thereafter. HUVEC with different CD109 expression were co-implanted with HCCLM3 or HepG2 cells in nude mice to investigate the effect of CD109 expression on tumor growth and metastasis. Reduced expression of CD109 on tumor vessels was associated with large tumor size, microvascular invasion, and advanced tumor stage. CD109 was an independent risk factor for disease-free survival (*P* = 0.001) after curative resection of HCC. CD109 knockdown in HUVEC promoted hepatoma cell proliferation, migration, and invasion. Interleukin-8 (IL-8) was a key tumor-promoting factor secreted from CD109 knockdown HUVEC. CD109 knockdown upregulated IL-8 expression through activation of TGF-β/Akt/NF-κB pathway in HUVEC. Co-implantation with CD109 knockdown HUVEC accelerated tumor growth and metastasis in mice models. In conclusion, CD109 expression on tumor vessels is a potential prognostic marker for HCC, and its reduced expression on TEC promoted tumor progression by paracrine IL-8.

## INTRODUCTION

Hepatocellular carcinoma (HCC) is a health threatening disease worldwide, with a high mortality [1]. Tumor metastasis is a leading cause of mortality for HCC [2]. However, the underlying mechanism has not been fully understood [3]. HCC is generally a highly vascularized malignancy with mainly blood-borne metastasis. Tumor-associated endothelial cells (TEC) are involved in this process, which may favor HCC progression [4–7]. In addition to forming channels to provide oxygen and nutrients for tumor development [8], TEC directly facilitate tumor progression by paracrine tumor-promoting cytokines [9–13]. Recent studies suggested that the potential of vascular endothelial cells (EC) to directly regulate the biological behavior of tumors likely transcends their structural roles in tumor vessels [14, 15]. However, the regulatory mechanism of TEC function is not clear.

CD109 is a glycosyl phosphatidylinositol–anchored glycoprotein which belongs to the α2-macroglobulin/complement superfamily and is located on the plasma membrane of platelets, activated T cells, and EC [16, 17]. Though expressed on several types of tumor cells [18], CD109 may be a potential TEC marker [19]. It was reported that CD109 was expressed on a small proportion of circulating TEC, which was used to predict the efficacy of anti-angiogenesis drugs in glioblastoma patients [20, 21]. However, CD109 could not discriminate tumor-derived circulating EC from normal circulating EC [22]. The role of CD109 in EC and its clinical significance in HCC patients have not been reported.

In this study, we aimed at clarifying whether CD109 was specifically expressed on TEC in HCC tissues. Furthermore, we investigated the correlation between CD109 expression on tumor vessels and the prognosis after curative resection of HCC. *In vitro* and *in vivo* experiments using human umbilical vein endothelial cells (HUVEC) were performed to study the effects of different CD109 expression on tumor growth and metastasis.

## RESULTS

### Reduced expression of CD109 on tumor vessels correlated with poor survival in HCC patients

**Figure 1:**
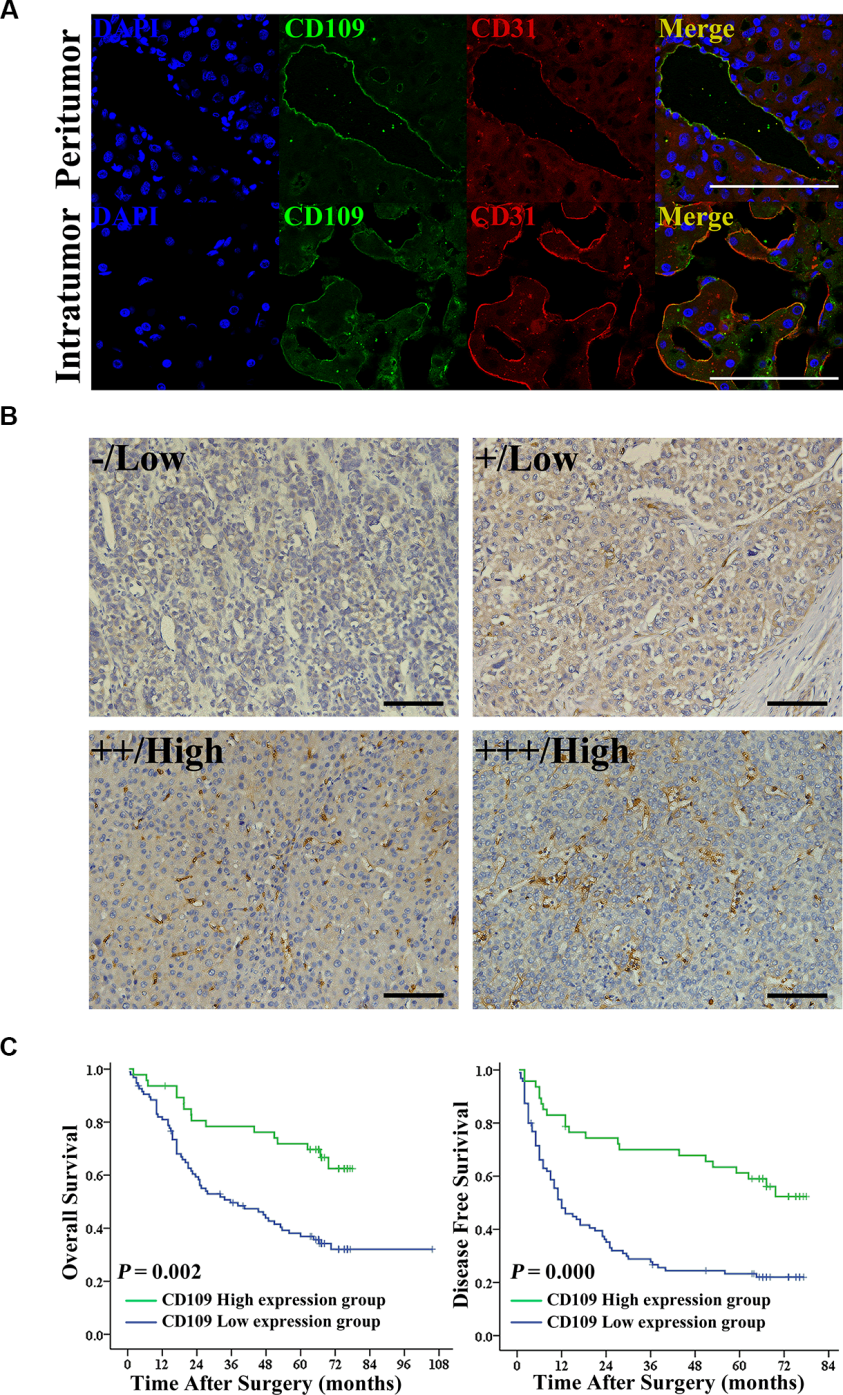
Reduced expression of CD109 on tumor vessels correlated with poor survival in HCC patients (**A**) Representative images of double immunofluorescence staining indicated that CD109 (green) co-localized with the CD31 (red) in the EC in HCC tissues. Cell nuclei were counterstained by DAPI (blue). (**B**) Representative images of immunohistochemistry staining indicated that CD109 was expressed on tumor vessels and the staining patterns were graded from 0 to +++. (**C**) Kaplan-Meier curves showed that reduced expression of CD109 on tumor vessels correlated with shorter overall survival and disease-free survival using log-rank test. Scale bars, 100 μm.

The double immunofluorescence staining showed that CD109 was co-localized with CD31 on EC, but it was not exclusively expressed on TEC in HCC tissues (Figure 1A). The immunohistochemistry staining of CD109 expression in a tissue microarray of 142 HCC patients showed that, in addition to tumor cells being positive for CD109 staining in a few patients (Supplementary Figure S1A), expression was mainly observed on tumor microvessels (Figure 1B). The patients were divided into low (*n* = 95) or high (*n* = 47) CD109 expression groups according to expression levels in tumor vessels (Figure 1B). The associations of CD109 expression in tumor vessels with clinicopathological characteristics were compared between the two groups (Supplementary Table S1). Patients with high CD109 expression on tumor vessels were older (*P* = 0.023), had smaller tumor size (*P* = 0.010), had less microvascular invasion (*P* = 0.036), and had earlier TNM stage (*P* = 0.015) than patients with low CD109 expression. Other characteristics, including sex, HBsAg, AFP, liver cirrhosis, tumor number, and tumor encapsulation, were not related to CD109 expression on tumor vessels. Patients in the low CD109 group experienced more recurrence and had shorter overall survival (Figure 1C). Moreover, multivariate analysis showed that low CD109 expression on tumor vessels was an independent risk factor for disease-free survival (*P* = 0.001) (Supplementary Table S2).

### CD109 expression was essential for EC function

**Figure 2:**
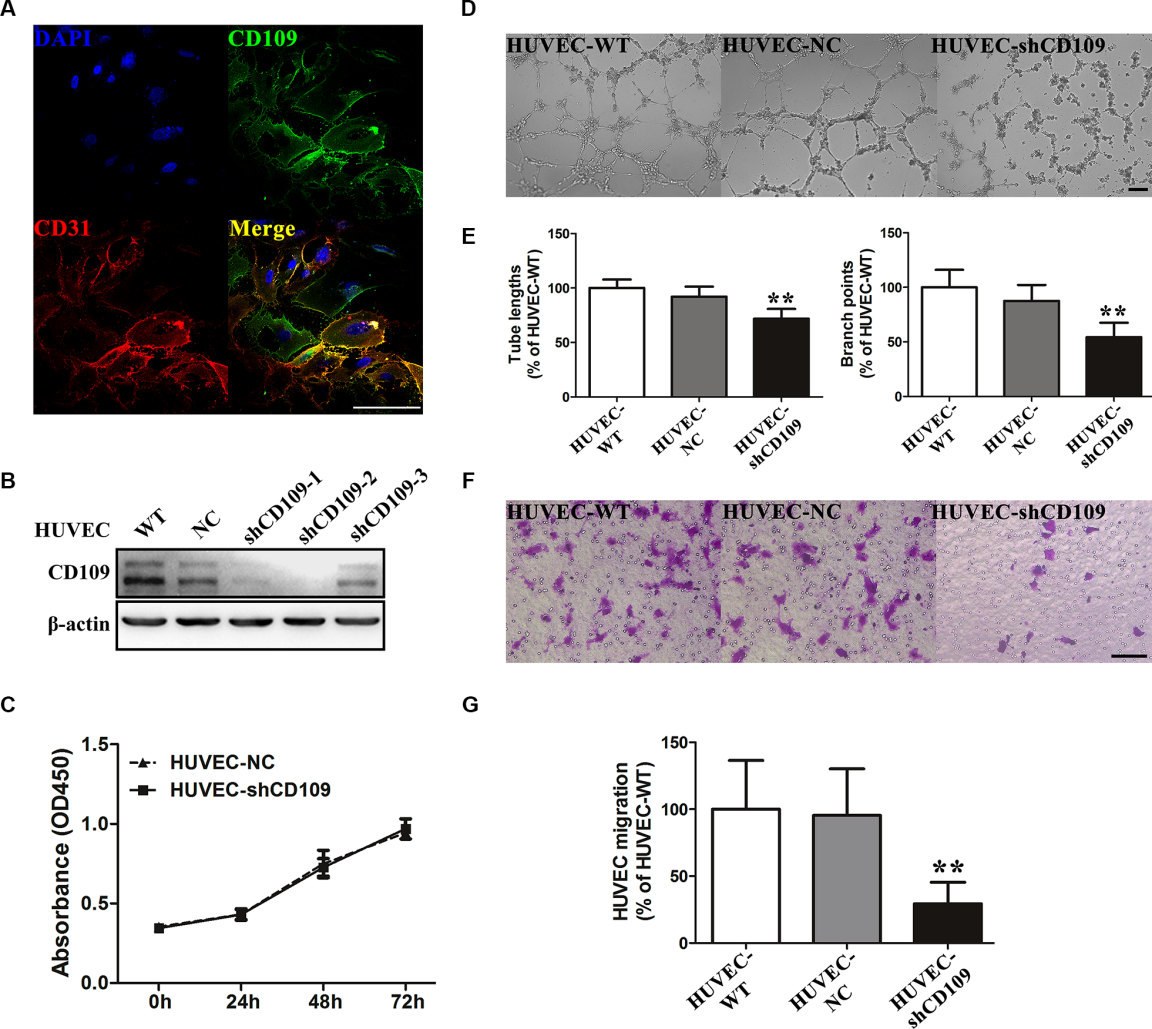
CD109 expression was essential for EC function (**A**) Representative images of double immunofluorescence staining CD109 (green) and CD31 (red) in HUVEC. (**B**) WB showed the silencing efficiency of CD109 in HUVEC after transduction with negative control shRNA (NC) or three different shCD109s. (**C**) Cell proliferation assay showed that CD109 knockdown did not affect HUVEC proliferation. (**D**) Representative images and quantitative data (**E**) showed that CD109 knockdown significantly decreased HUVEC total tube lengths and branch points in the tube formation assay on Matrigel. (**F**) Representative images and quantitative data (**G**) showed that CD109 knockdown in HUVEC significantly inhibited cell migration in the Boyden chamber assay. Scale bars, 100 μm. β-actin served as a loading control for WB. Data shown as mean ± standard deviation (SD) were from triplicates of three independent experiments. *P < 0.05; **P < 0.01 by ANOVA. WT, wild type; NC, negative control.

Sporadic expression of CD109 can be detected on a few human hepatoma cell lines, and it is highly expressed on HUVEC by quantitative real-time PCR (qRT-PCR), Western Blotting (WB), and immunofluorescence staining (Figure 2A; Supplementary Figure S2A–S2C). WB showed that three different small hairpin RNA (shRNA) exhibited different efficiency on suppression of CD109 expression in HUVEC (Figure 2B). We chose the most robust inhibitory CD109 shRNA (shCD109) in our study. CD109 knockdown did not change HUVEC proliferation (Figure 2C), but it inhibited EC tube formation on Matrigel, as judged by total tube lengths and branch points (Figure 2D, 2E), and suppressed cell migration (Figure 2F, 2G).

### CD109 knockdown in HUVEC enhanced paracrine effects on hepatoma cells proliferation, migration, and invasion *in vitro*

**Figure 3:**
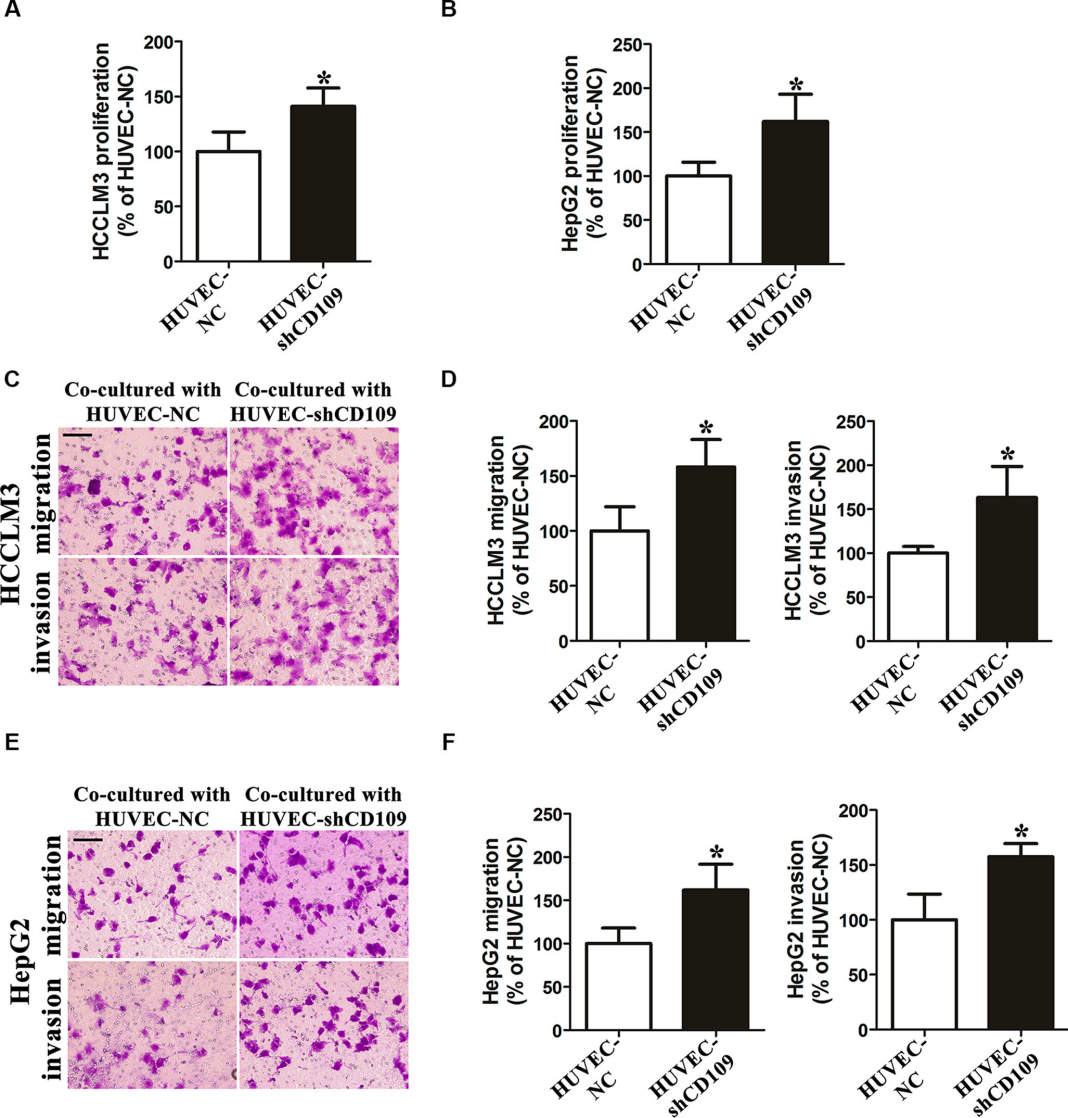
CD109 knockdown in HUVEC enhanced paracrine effects on hepatoma cells proliferation, migration, and invasion *in vitro* (**A**–**B**) Cell proliferation assay showed HCCLM3 and HepG2 cells proliferations were significantly increased when co-cultured with HUVEC-shCD109 compared with HUVEC-NC at 72 h. (**C**) Representative images and (**D**) quantitative data of Boyden chamber cell migration and invasion assays showed that HUVEC-shCD109 promoted HCCLM3 cells migration and invasion as compared with HUVEC-NC. (**E**) Representative images and (**F**) quantitative data of Boyden chamber cell migration and invasion assays showed that HUVEC-shCD109 promoted HepG2 cells migration and invasion as compared with HUVEC-NC. Scale bars, 100 μm. Data shown as mean ± SD were from triplicates of three independent experiments. *P < 0.05 by *t* test. NC, negative control.

HCCLM3 and HepG2 cells proliferations were significantly increased when co-cultured with HUVEC-shCD109 compared with HUVEC-NC at 72 h (Figure 3A, 3B). Cell migration and invasion assays showed that the number of migrated and invaded HCCLM3 and HepG2 cells was significantly increased when co-cultured with HUVEC-shCD109 compared with HUVEC-NC (Figure 3C–3F).

### IL-8 mediated the tumor-promoting role of CD109 knockdown in HUVEC

**Figure 4:**
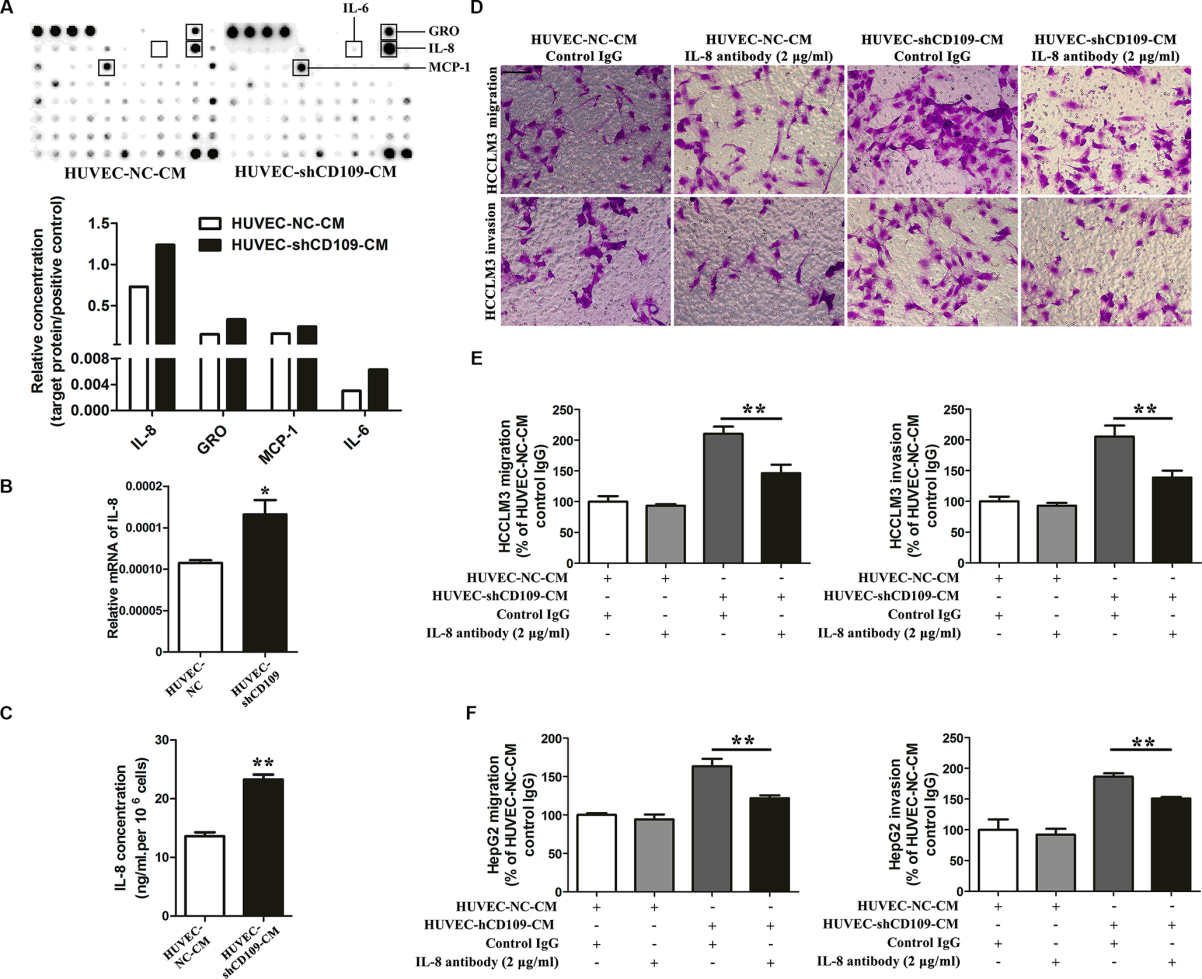
IL-8 mediated the tumor-promoting role of CD109 knockdown in HUVEC (**A**) Human cytokine antibody array assays showed that elevated levels of four cytokines were identified in the CM of HUVEC-shCD109 compared with HUVEC-NC. (**B**) qRT-PCR showed that the mRNA expression of IL-8 was increased in HUVEC-shCD109 compared with HUVEC-NC. (**C**) ELISA showed that CD109 knockdown HUVEC secreted a significantly higher level of IL-8 compared with HUVEC-NC. (**D**) Representative images and (**E**) quantitative data of Boyden chamber cell migration and invasion assays showed that HCCLM3 cells migration and invasion promoted by CD109 knockdown were significantly suppressed when IL-8 was neutralized. (**F**) Quantitative data of Boyden chamber cell migration and invasion assays showed that HepG2 cells migration and invasion promoted by CD109 knockdown were significantly suppressed when IL-8 was neutralized. Data shown as mean ± SD were from triplicates of three independent experiments. *P < 0.05; **P < 0.01 by *t* test. CM, conditioned media; NC, negative control.

Human cytokine antibody array was used to screen the key tumor-promoting factor in the conditioned media (CM) of HUVEC. Elevated levels of four cytokines were identified (Figure 4). We selected IL-8 for validation. qRT-PCR showed that IL-8 mRNA expression increased in HUVEC-shCD109 (Figure 4B). ELISA also confirmed that the level of IL-8 was higher in the CM of HUVEC-shCD109 compared with that of HUVEC-NC (Figure 4C). IL-8 neutralizing antibody was used to verify the key role of IL-8. Neutralization of IL-8 resulted in a partial but significant inhibition of HUVEC-shCD109–mediated promotion of tumor cell migration and invasion (Figure 4D–4F; Supplementary Figure S3A); however, it had limited effect on hepatoma cells proliferations (Supplementary Figure S3B).

### CD109 knockdown upregulated IL-8 expression through activation of TGF-β/Akt/NF-κB pathway in HUVEC

**Figure 5:**
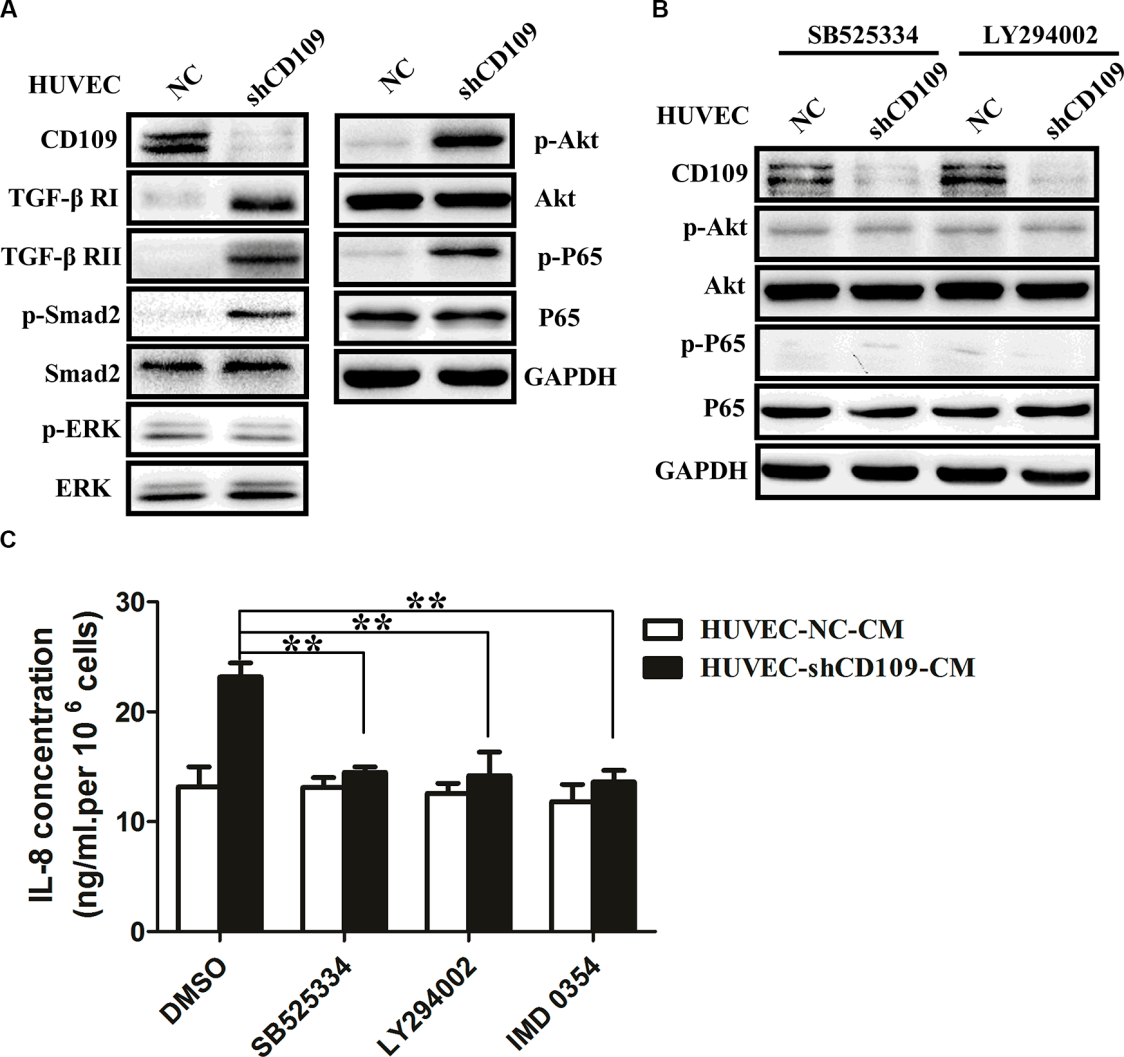
CD109 knockdown upregulated IL-8 expression through activation of TGF-β/Akt/NF-κB pathway in HUVEC (**A**) WB showed that CD109 knockdown significantly increased protein expression of TGF-β RI, TGF-β RII, p-Smad2, p-Akt, and p-P65 in HUVEC. (**B**) WB showed that SB525334 or LY294002 significantly decreased protein expression of p-Akt and p-P65 in CD109 knockdown HUVEC. (**C**) ELISA showed that the elevated IL-8 was significantly inhibited in HUVEC-shCD109 when pretreated with SB525334, LY294002, or IMD 0354. GAPDH served as a loading control for WB. Data shown as mean ± SD were from triplicates of three independent experiments. **P < 0.01 by *t* test. CM, conditioned media; NC, negative control.

WB revealed that CD109 knockdown significantly increased expression of TGF-β receptor I (TGF-β RI), TGF-β receptor II (TGF-β RII), p-Smad2, p-Akt, and p-P65 in HUVEC. However, we did not observe any change in Smad2, Akt, P65, p-ERK, and ERK expression (Figure 5A). TGF-β pathway inhibitor SB525334 or PI3K/Akt pathway inhibitor LY294002 decreased expression of p-Akt and p-P65 in CD109 knockdown HUVEC (Figure 5B). Furthermore, SB525334, LY294002, or NF-κB inhibitor IMD 0354 significantly decreased the elevated IL-8 in HUVEC-shCD109 (Figure 5C).

### CD109 knockdown in HUVEC facilitated tumor growth and metastasis *in vivo*

**Figure 6:**
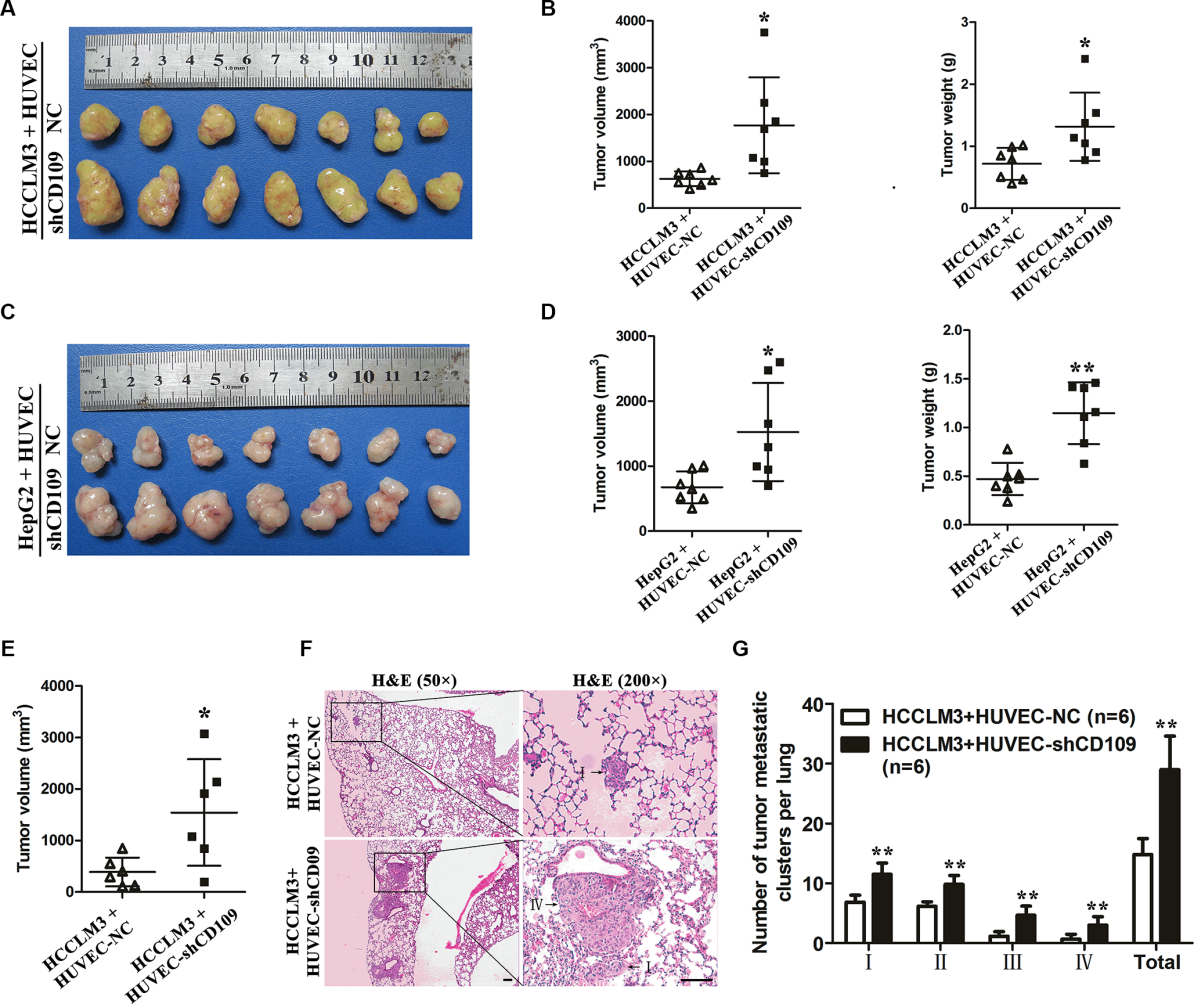
CD109 knockdown in HUVEC facilitated tumor growth and metastasis *in vivo* (**A**) Macrograph of subcutaneous tumors derived from HCCLM3 co-implantation with HUVEC-NC or HUVEC-shCD109. (**B**) Tumor volume and weight of HCCLM3-HUVEC co-implantation tumors were compared between the two groups (*N* = 7 mice per group). (**C**) Macrograph of subcutaneous tumors derived from HepG2 co-implantation with HUVEC-NC or HUVEC-shCD109. (**D**) Tumor volume and weight of HepG2-HUVEC co-implantation tumors were compared between the two groups (*N* = 7 mice per group). (**E**) Tumor volumes of HCCLM3-HUVEC co-implantation tumors were compared between the two groups in an orthotopic mouse model (*N* = 6 mice per group). (**F**) Representative images of lung metastatic foci with magnification of the selected areas (hematoxylin & eosin) were shown. Arrows indicated tumor grades. (**G**) The number and grade of lung metastatic foci were compared between the two groups (*N* = 6 mice per group). Scale bars, 100 μm. Data are presented as mean ± SD. *P < 0.05; **P < 0.01 by *t* test. NC, negative control.

In a subcutaneous mouse model, HCCLM3 co-implanted with HUVEC-shCD109 yielded larger tumor volume and higher tumor weight than those co-implanted with HUVEC-NC (volume: 1769.4 ± 1024.9 mm^3^ vs. 629.4 ± 157.3 mm^3^, *P* = 0.026; weight: 1.32 ± 0.55 g vs. 0.72 ± 0.26 g, *P* = 0.023) (Figure 6A, 6B). Consistent results were observed when we used HepG2 cells (Figure 6C, 6D). In an orthotopical mouse model, HCCLM3 co-implanted with HUVEC-shCD109 yielded larger tumor volume than those co-implanted with HUVEC-NC (1542.2 ± 1034.8 mm^3^ vs. 388. 3 ± 280.4 mm^3^; *P* = 0.04) (Figure 6E). Although the incidence of lung metastasis was 100% in two groups, the number and the grade of metastatic clusters per lung were much greater in HUVEC-shCD109 group than in NC group (Figure 6F, 6G).

## DISCUSSION

Previous studies focused more on CD109 expression on tumor cells and its associations with malignant behavior of tumor cells and patient prognosis [18, 23–29]. In this study, we found that CD109 was also expressed on HCC tumor cells, but its expression on tumor cells was not associated with patient prognosis (Supplementary Figure S1B). Several studies suggested that CD109 expression on circulating TEC may have potential clinical values [20, 21]. However, the expression of CD109 in tumor vessels and its clinical significance were not mentioned. In this study, we found that reduced expression of CD109 on tumor vessels was correlated with large tumor size, microvascular invasion, advanced tumor stage, and poor survival after curative resection of HCC. It is speculated that reduced expression of CD109 on TEC may facilitate tumor growth and metastasis in HCC patients.

In the present study, we found that CD109 knockdown inhibited tube-forming capability of HUVEC, but significantly promoted tumor growth and metastasis in a paracrine manner. Our results supported the notion that the paracrine tumor-promoting effects of TEC may surpass its passive role in tumor angiogenesis [14, 15]. As dysfunctional EC, TEC secreted a wide range of growth factors and cytokines to promote tumor progression [14]. Franses et al. revealed that Perlecan knockdown in EC significantly increased IL-6 secretion which accelerated the invasiveness of tumor cells [15]. A recent study showed that AIP1-deficient EC induced epithelial–mesenchymal transition (EMT) of tumor cells by the secretion TGF-β2 [13]. In the present study, we first verified that the tumor-promoting effects of CD109 knockdown in HUVEC were mainly mediated by paracrine IL-8. IL-8, which is a negative prognostic marker for HCC [30, 31], promotes tumor angiogenesis and metastasis [32]. As is reported, IL-8 could be secreted from both tumor cells and certain stromal cells [33, 34]. The upregulation of IL-8 in CD109 knockdown HUVEC suggests that TEC may be an important source of IL-8 in HCC microenvironment. A previous study also showed that TEC with Bcl-2 overexpression in head and neck cancer patients promoted tumor cell proliferation and invasion by paracrine IL-8 [9]. We postulated that IL-8 secreted by TEC may facilitate the transendothelial migration of tumor cells.

As a co-receptor of TGF-β, CD109 inhibited TGF-β pathway through TGF-β receptors internalization and degradation in keratinocytes [35–37]. In the present study, we first revealed that CD109 knockdown significantly increased the TGF-β receptors expression, and then constitutively activated the TGF-β pathway and its downstream Smad-dependent and Smad-independent PI3K/Akt/NF-κB pathway in HUVEC. We further verified that CD109 knockdown upregulated IL-8 expression through activation of TGF-β/Akt/NF-κB pathway in HUVEC. These results were consistent with previous studies showing that activation of TGF-β, Akt, and NF-κB pathways upregulated the IL-8 expression in EC [38–40].

In conclusion, CD109 expression on tumor vessels serves as a prognostic marker for HCC patients after curative resection. Suppressing tumor-promoting cytokines secretion from TEC may have potential clinical value in the prevention of HCC metastasis.

## MATERIALS AND METHODS

### Patients and follow-up

From March 2004 to December 2006, archived specimens for tissue microarray construction from 142 consecutive patients who underwent curative resection for HCC by the same surgical team in our institute (Liver Cancer Institute, Fudan University) were collected. None of the patients received any anti-cancer therapy before surgery. The criteria for curative resection were defined as macroscopically complete removal of the tumor as described previously [41]. Tumor stage was determined according to the 7th edition of AJCC/UICC TNM classification system [42]. Tumor differentiation was graded by the Edmondson grading system. Follow-up procedures and postoperative treatment modalities were carried out according to a uniform guideline as described in our previous study [41]. The median follow-up period was 39.0 months (range, 1.5–95.0). Disease-free survival or overall survival was defined as the interval between the date of surgery and the date of recurrence or death; data were censored on the date of last follow-up for patients without recurrence or death. This study was approved by the Zhongshan Hospital Research Ethics Committee and informed consent was obtained from each patient according to the committee's regulations.

### Cell culture

Human HCC cell lines MHCC97L, MHCC97H, HCCLM3 (with various metastatic potential, established in our institute), SMMC7721, Huh-7, PLC/PRF/5, Hep3B, and HepG2 (Chinese Academy of Sciences) were cultured in Dulbecco's modified Eagle's medium (Invitrogen, Carlsbad, CA, USA) containing 10% fetal bovine serum (Invitrogen, Carlsbad, CA, USA), 100 IU/mL penicillin G and 100 mg/mL streptomycin sulfate (Sigma-Aldrich, St. Louis, MO, USA) in a humidified atmosphere of 5% CO_2_ at 37°C. Human normal liver L-02 cells (Chinese Academy of Sciences) were cultured under the same conditions. Primary HUVEC (AllCells, Shanghai, China) were cultured in endothelial cell growth medium-2 (Lonza, Basel, Switzerland) supplemented with 10% fetal bovine serum and used within two to six passages.

### Cell transfection

For permanent inhibition of CD109 expression in HUVEC, we constructed recombinant lentivirus vectors containing small hairpin RNA (shRNA) targeting human CD109. These vectors and the negative control vector were transfected into HEK-293T cells using Lipofectamine 2000 transfection reagent (Invitrogen, Carlsbad, CA, USA). The vectors used for CD109 knockdown were U6-MCS-ubiquitin-Cherry-IRES-puromycin (Genechem, Shanghai, China). HUVEC were then transfected with lentivirus following the manufacturer's instructions, and WB was used to validate the efficiency of CD109 knockdown. The most effective oligonucleotides for CD109 (5ʹ-GACACUUACUCUUCCAUCA-3ʹ, sense) that almost completely silenced its expression were selected for the study.

### Cell proliferation, migration and invasion assays

Cell proliferation was assayed using CCK-8 solution (Dojindo, Kumamoto, Japan). Cell migration assays were performed using Boyden chambers with an 8-μm pore size (Corning, Tewksbury, MA, USA) as described previously [43]. Cell invasive assays were performed similarly except that filters pre-coated with Matrigel (BD Biosciences, San Jose, CA, USA) were used and the incubation time was 48 h. Three independent experiments were performed in triplicate.

### Tube formation assay

The 96-well plates (Corning, Tewksbury, MA, USA) were pre-coated with growth factor reduced Matrigel (BD Biosciences, San Jose, CA, USA) for 30 min at 37°C and HUVEC (1.5 × 10^4^ cells /well) were re-suspended in endothelial cell basal medium-2 (EBM-2; Lonza, Basel, Switzerland) and plated into each well. Tube formation was visualized and photographed under an inverted microscope (Olympus, Tokyo, Japan) after 6 h and analyzed by counting the total number of branch points and total tube lengths from five random fields at 100 × magnification using ImageJ software (National Institutes of Health, Bethesda, MD, USA). Three independent experiments were performed in triplicate.

### Hepatoma cells proliferation, migration, and invasion assay in HUVEC-Hepatoma cells co-culture system

Hepatoma cell proliferation assay in the co-culture system, HCCLM3 or HepG2 cells (30 000 cells/well) were cultured in the lower wells of 24-well plates in the EBM-2 supplemented with 1% FBS. Meanwhile, HUVEC with or without CD109 knockdown (30 000 cells/well) was plated onto inserts (0.4-μm pore size) in the EBM-2 supplemented with 1% FBS. The inserts were carefully layered onto the companion plates for co-culturing. After 72 h, inserts were removed and hepatoma cells were trypsinized and counted using a hemocytometer. Hepatoma cell migration and invasion assay in the co-culture system, HUVEC (50 000 cells/well) was cultured in the lower wells in the EBM-2 supplemented with 1% FBS. Hepatoma cells (50 000 cells/well) were plated onto inserts (8-μm pore size) pre-coated with or without growth factor reduced Matrigel in serum-free EBM-2, and then the inserts were carefully layered onto the companion plates for 24 h or 48 h. Three independent experiments were performed in triplicate.

### Collection of conditioned media of HUVEC

As previously described [11], CM was collected from confluent HUVEC by 24 h of culture in serum-free EBM-2. The same medium was incubated for 24 h without HUVEC to serve as basal medium. Cells and debris were removed by centrifugation at 3000 rpm for 20 min at 4°C. CM were then filtered through 0.22-μm filters and stored at −80°C for further use. The protein concentration of the CM was measured by bicinchoninic acid protein assay (Beyotime Biotechnology, Shanghai, China). HUVEC were pre-treated with SB525334 (1 μM; Selleckchem, Houston, TX, USA), LY294002 (20 μM; Cell Signaling Technology, USA), IMD 0354 (10 μM; Selleckchem, Houston, TX, USA), or DMSO (0.1%; Sigma-Aldrich, St. Louis, MO, USA) for 12 h, and then incubated in EBM-2 for 24 h to collect CM.

### Human cytokine antibody array and IL-8 ELISA

The amounts of secreted cytokines were compared between HUVEC with and without CD109 knockdown using C-Series Human Cytokine Antibody Array C5 for 80 human cytokines (RayBiotech, Norcross, GA, USA) as directed by the manufacturer. The concentration of IL-8 in the CM of HUVEC was detected by ELISA (Anogen, YES Biotech, Ontario, Canada) according to the manufacturer's instructions and normalized by volume and cell number. To validate the role of IL-8, 2 μg/mL neutralizing antibody of IL-8 (R&D Systems, Wiesbaden, Germany) was added to the CM to neutralize the IL-8. The same concentration of isotype control immunoglobulin G (IgG) antibody (R&D Systems, Wiesbaden, Germany) was used as control.

### RNA isolation and qRT-PCR

RNA isolation and qRT-PCR procedures have been described previously [43]. The primers for amplification of human genes were as follows: CD109, forward 5ʹ-CCTGTGACCTTTGCAGTGATGT-3ʹ and reverse 5ʹ-GAGTGATGATGGGAGCCTGAA-3ʹ; IL-8, forward 5ʹ-CAGCCTTCCTGATTTCTGC-3ʹ and reverse 5ʹ-GGGTGGAAAGGTTTGGAGTA-3ʹ; and glyceraldehyde-3-phosphate dehydrogenase (GAPDH), forward 5ʹ-TGACTTCAACAGCGACACCCA-3ʹ and reverse 5ʹ-CACCCTGTTGCTGTAGCCAAA-3ʹ. All samples were performed in triplicate three times and GAPDH values were used to normalize gene expression.

### Western blotting

Procedures are described elsewhere [43]. Primary antibodies were listed in the Supplementary Table S3.

### Immunofluorescence staining and confocal microscopy

Procedures are described in our previous study [43]. Primary antibodies were listed in the Supplementary Table S3.

### Tissue microarray and immunohistochemistry staining

As previously described [44], a tissue microarray was constructed and used to assess the expression of CD109 and its prognostic significance. Immunohistochemistry staining was conducted as described in our previous study [45]. Primary antibody was listed in the Supplementary Table S3. The immunohistochemical intensity of CD109 staining was evaluated by two independent senior pathologists without knowledge of patient outcome, and discrepancies were resolved by discussion. The density of positive staining was scored using a 4-point scale (0–+++) based on the amount of positive tumor vasculature and the intensity of the staining. Briefly, no staining was recorded as –, weak staining was recorded in fewer than one-third of the regions as +, moderate staining was recorded in one-third to two-thirds of the regions as ++, and strong staining was recorded in more than two-thirds of the section as +++. The patients were dichotomized to low (− and +) or high (++ and +++) CD109 expression groups.

### Xenograft model in nude mice

Approximately 5 × 10^6^ HCCLM3 or HepG2 cells mixed with the same number of HUVEC with or without CD109 knockdown were rinsed twice with PBS to remove serum and trypsin after harvesting, re-suspended in serum-free Dulbecco's modified Eagle's medium and injected subcutaneously into the right front flanks of the nude mice (seven in each group, total of four groups). After 4 weeks, the mice were killed under anesthesia and tumor samples were dissected. Tumor volumes (largest diameter × perpendicular height^2^/2) and weights were measured. Similarly, to observe differences in lung metastasis, 2 × 10^6^ HCCLM3 cells and the same number of HUVEC with or without CD109 knockdown were re-suspended in 40 μL serum-free Dulbecco's modified Eagle's medium/Matrigel (1:1; BD Biosciences, San Jose, CA, USA) and orthotopically injected into the left hepatic lobe of each mouse (six in each group, total of two groups). After 6 weeks, mice were sacrificed, tumor volumes were measured, and their lungs were dissected and fixed with 4% neutral buffered formalin. The total number of lung metastases was counted as described previously [46]. Briefly, the first of 10 of every 100 serial sections per lung were stained with hematoxylin and eosinand calculated independently by two pathologists in a blinded manner. The metastases were classified into four grades based on the number of tumor cells present at the maximal section for each metastatic lesion: grade I, ≤ 20 tumor cells; grade II, 20 to 50 tumor cells; grade III, 50 to 100 tumor cells; and grade IV, > 100 tumor cells.

### Statistical analysis

Data were analyzed using SPSS 16.0 for Windows (SPSS Inc., Chicago, IL, USA). The Pearson χ^2^ test or Fisher's exact test was used to compare categorical variables. Quantitative variables expressed as mean ± SD were analyzed by one-way ANOVA or Student's *t* test. The correlation between CD109 expression and survival of patients was assessed using the Kaplan-Meier curves and compared by log-rank test. The Cox proportional hazards regression model was used to perform univariate and multivariate analyses. *P* < 0.05 was considered statistically significant.

## SUPPLEMENTARY MATERIALS FIGURES AND TABLES



## Abbreviations

CM: conditioned medium
EC: endothelial cells
ELISA: enzyme-linked immunosorbent assay
HCC: hepatocellular carcinoma
HUVEC: human umbilical vein endothelial cells
IL-8: interleukin-8
qRT-PCR: quantitative real-time polymerase chain reaction
shRNA: small hairpin ribonucleic acid
TEC: tumor-associated endothelial cells
TGF-β: transforming growth factor-β
WB: western blotting.
