# Supplementary material for: Reduced expression of CD109 in tumor-associated endothelial cells promotes tumor progression by paracrine interleukin-8 in hepatocellular carcinoma
Source: Oncotarget. 2016 Apr 18;7(20):29333–45. doi: 10.18632/oncotarget.8787 (PMC5045399; doi:10.18632/oncotarget.8787)
Supplement: Supplementary file 1 [file oncotarget-07-29333-s001.pdf]

Reduced expression of CD109 in tumor-associated endothelial cells promotes tumor progression by paracrine interleukin-8 in hepatocellular carcinoma

Supplementary Materials

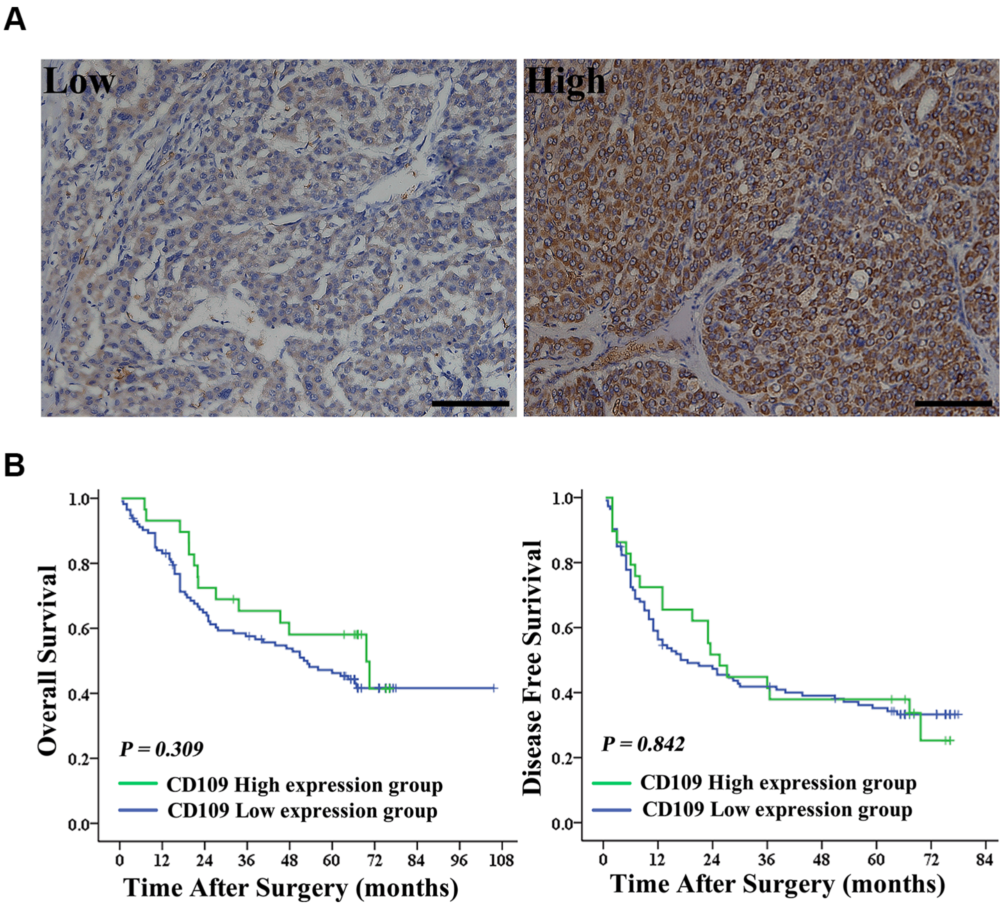

Supplementary Figure S1: The expression of CD109 on HCC tumor cells and its association with patient prognosis. (**A**) Representative images of IHC staining showed the expression of CD109 on HCC tumor cells. (**B**) Kaplan-Meier curves showed that CD109 expression on HCC tumor cells was not associated with overall survival and disease-free survival using log-rank test. Scale bars, 100 μm.

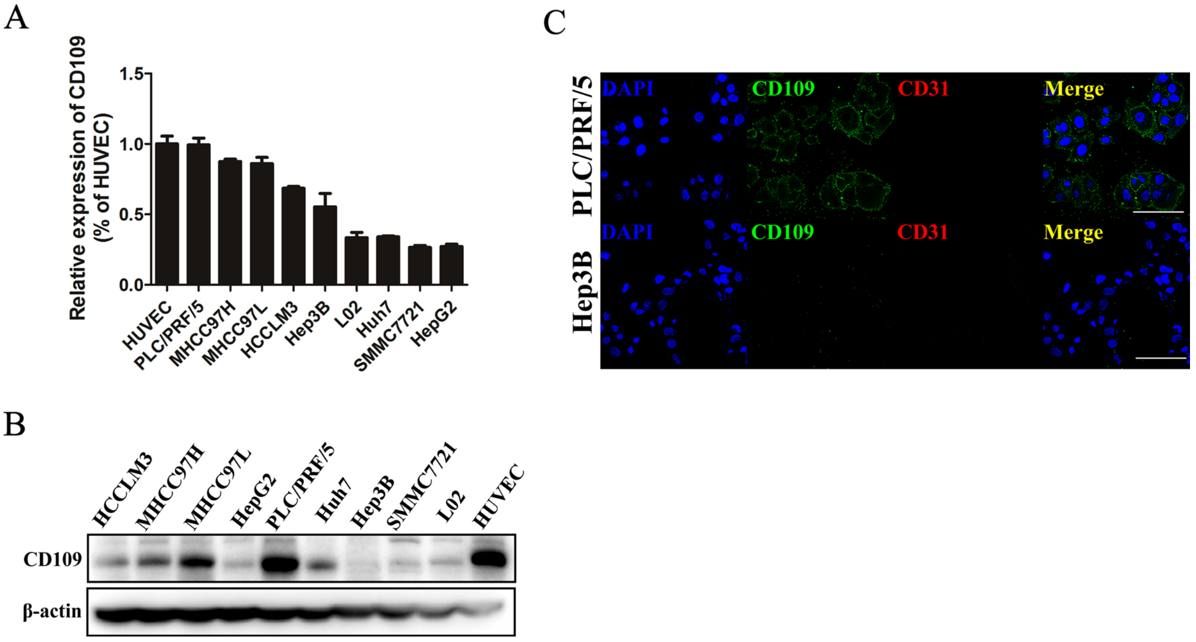

Supplementary Figure S2: The expression of CD109 on several cell lines. (**A**) qRT-PCR and (**B**) WB showed the different CD109 mRNA and protein expression in eight human HCC cell lines and the human normal liver cell line (L02) and HUVEC. (**C**) Representative images of double IF staining CD109 (green) and CD31 (red) in PLC/PRF/5 and Hep3B cells. Scale bars, 100 μm. GAPDH was used as internal control for qRT-PCR. β-actin served as a loading control for WB. Data shown as mean ± SD were from triplicates of three independent experiments.

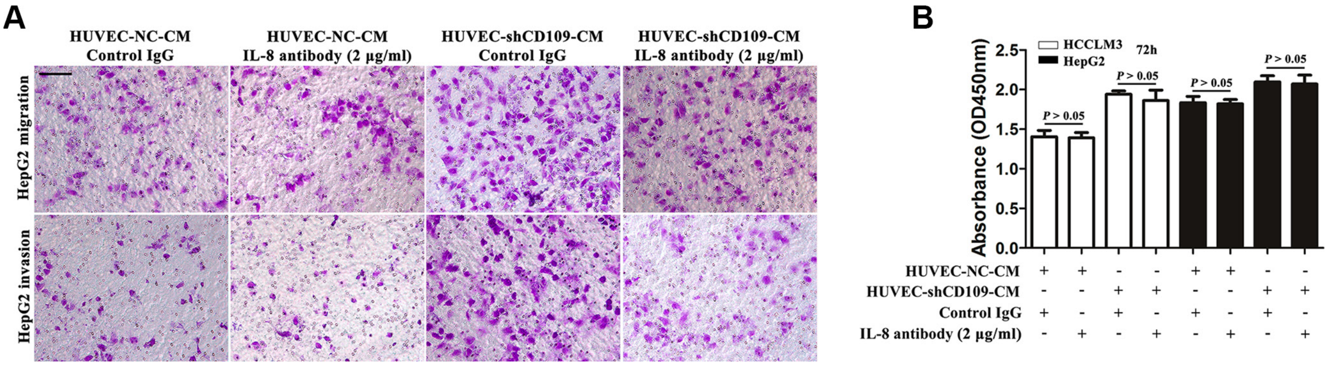

Supplementary Figure S3: IL-8 mediated the paracrine pro-tumor effects of CD109 knockdown in HUVEC. (**A**) Representative images of Boyden chamber cell migration and invasion assays showed that HepG2 cell migration and invasion promoted by CD109 knockdown were significantly suppressed when IL-8 was neutralized. (**B**) Cell proliferation assays showed that neutralization of IL-8 did not inhibit HUVEC-shCD109 mediated promotion of tumor cell proliferation. Scale bars, 100 μm. Data shown as mean ± SD were from triplicates of three independent experiments. $P > 0.05$ by t test. CM, conditioned media; NC, negative control.

Supplementary Table S1: Correlation between CD109 expression on tumor vessels and clinicopathological characteristics

	CD109 expression on tumor vessels (n = 142)				
**Variable**	**High (n = 47)**		**Low (n = 95)**		
	**No. of patients**	**%**	**No. of patients**	**%**	***P* value**
Age, years[a]	54.0 ± 9.9		49.6 ± 11.1		**0.023**
Gender					0.615
Male	38	80.9	80	84.2	
Female	9	19.1	15	15.8	
HBsAg					0.787
Positive	43	91.5	84	88.4	
Negative	4	8.5	11	11.6	
AFP					0.373
≤ 20 ng/ml	26	55.3	45	47.4	
> 20 ng/ml	21	44.7	50	52.6	
Liver cirrhosis					
Yes	40	85.1	73	76.8	0.250
No	7	14.9	22	23.2	
Tumor size					**0.010**
≤ 5 cm	34	72.3	47	50.5	
> 5 cm	13	27.7	48	49.5	
Tumor number					0.179
Single	43	91.5	79	83.2	
Multiple	4	8.5	16	16.8	
Tumor encapsulation					0.065
Complete	27	57.4	39	41.1	
No	20	42.6	56	58.9	
Microvascular invasion					**0.036**
Yes	15	31.9	48	50.5	
No	32	68.1	47	49.5	
Tumor differentiation					0.114
I–II	35	74.5	58	61.1	
III–IV	12	25.5	37	38.9	
TNM stage					**0.015**
I	29	61.7	38	40.0	
II	16	34.0	39	41.1	
IIIA	2	4.3	18	18.9	

Abbreviations: HBsAg, hepatitis B s antigen; AFP, α-fetoprotein; TNM, tumor-nodes-metastasis.
[a]Student's *t*-test.

Supplementary Table S2: Univariate and multivariate analyses of factors associated with survival and recurrence

	OS				DFS			
		Multivariate				Multivariate		
**Features**	**Univariate *P***	**HR**	**95% CI**	***P***	**Univariate *P***	**HR**	**95% CI**	***P***
Age: < 52 vs ≥ 52 years	0.809			NA	0.779			NA
Gender: female vs male	0.367			NA	0.332			NA
HBsAg: positive vs negative	0.320			NA	0.164			NA
Liver cirrhosis: yes vs no	0.230			NA	0.302			NA
AFP: > 200 ng/dLvs ≤ 200 ng/dL	0.447			NA	0.428			NS
Tumor size: > 5 cm vs ≤ 5 cm	**0.000**	2.605	1.383–4.909	**0.003**	**0.002**			NS
Tumor number: single vs multiple	0.559			NA	**0.024**			NS
Tumor encapsulation: complete vs none	0.317			NA	0.491			NA
Microvascular invasion: no vs yes	**0.006**			NS	**0.041**			NS
Tumor differentiation: III–IV vs I–II	**0.001**			NA	**0.000**	**2.451**	**1.410–4.260**	**0.001**
TNM stage: IIIA vs II vs I	**0.000**			NS	**0.001**	**2.540**	**1.107–5.830**	**0.028**
Tumor vessels CD109 high vs low	**0.002**	0.487	0.234–1.014	0.054	**0.000**	**0.322**	**0.166–0.623**	**0.001**

Abbreviations: OS, overall survival; DFS, disease-free survival; HR, hazard ratio; CI, confidence interval; NA, not adopted; NS, not significant; HBsAg, hepatitis B s antigen; AFP, α-fetoprotein; NS, not significant; TNM, tumor-nodes-metastasis.

Supplementary Table S3: Primary antibodies for WB, IHC and IF

Primary antibody	Concentration for WB	Concentration for IHC	Concentration for IF	Specificity	Molecular weight	Company
CD109		1:100		Mouse monoclonal	159	R&D
CD109	1:2000		1:100	Sheep polyclonal	159	R&D
CD31			1:50	Mouse monoclonal	130	Santa Cruz
TGF-β RI	1:1000			Rabbit polyclonal	56	Abcam
TGF-β RII	1:500			Rabbit polyclonal	65	Abcam
p-Smad2	1:5000			Rabbit monoclonal	52	Abcam
Smad2	1:5000			Rabbit monoclonal	52	Abcam
Akt	1:1000			Rabbit monoclonal	60	Cell Signaling Technology
p-Akt	1:1000			Rabbit monoclonal	60	Cell Signaling Technology
p-ERK	1:1000			Rabbit monoclonal	44/42	Cell Signaling Technology
ERK	1:1000			Rabbit monoclonal	44/42	Cell Signaling Technology
P65	1:5000			Rabbit monoclonal	65	Abcam
p-P65	1:5000			Rabbit monoclonal	65	Abcam
β-actin	1:5000			Mouse monoclonal	43	Sigma
GAPDH	1:5000			Mouse monoclonal	36	Cell Signaling Technology

Abbreviations: WB, western blotting; IHC, immunohistochemistry; IF, immunofluorescence.
